# Performance of Serum C-Reactive Protein as a Screening Test for Smear-Negative Tuberculosis in an Ambulatory High HIV Prevalence Population

**DOI:** 10.1371/journal.pone.0015248

**Published:** 2011-01-10

**Authors:** Douglas Wilson, Motasim Badri, Gary Maartens

**Affiliations:** 1 Department of Medicine, Edendale Hospital, Pietermaritzburg, University of KwaZulu-Natal, KwaZulu-Natal, South Africa; 2 Department of Medicine, University of Cape Town, Cape Town, South Africa; 3 Division of Clinical Pharmacology, Department of Medicine, University of Cape Town, Cape Town, South Africa; McGill University, Canada

## Abstract

**Background:**

Delayed diagnosis has contributed to the high mortality of sputum smear-negative tuberculosis (SNTB) in high HIV prevalence countries. New diagnostic strategies for SNTB are urgently needed. C-reactive protein (CRP) is a non-specific inflammatory protein that is usually elevated in patients with tuberculosis, but its role in the diagnosis of tuberculosis is uncertain.

**Methodology/Principal Findings:**

To determine the diagnostic utility of CRP we prospectively evaluated the performance of CRP as a screening test for SNTB in symptomatic ambulatory tuberculosis suspects followed up for 8 weeks in KwaZulu-Natal, South Africa. Confirmed tuberculosis was defined as positive culture or acid-fast bacilli with granulomata on histology, and possible tuberculosis as documented response to antitubercular therapy. The CRP quotient was defined as a multiple of the upper limit of normal of the serum CRP result. Three hundred and sixty four participants fulfilled entry criteria: 135 (37%) with confirmed tuberculosis, 114 (39%) with possible tuberculosis, and 115 (24%) without tuberculosis. The median CRP quotient was 15.4 (IQR 7.2; 23.3) in the confirmed tuberculosis group, 5.8 (IQR 1.4; 16.0) in the group with possible tuberculosis, and 0.7 (IQR 0.2; 2.2) in the group without tuberculosis (*p*<0.0001). The CRP quotient above the upper limit of normal had sensitivity 0.98 (95% CI 0.94; 0.99), specificity 0.59 (95% CI 0.50; 0.68), positive predictive value 0.74 (95% CI 0.67; 0.80), negative predictive value 0.96 (95% CI 0.88; 0.99), and diagnostic odds ratio 63.7 (95% CI 19.1; 212.0) in the confirmed tuberculosis group compared with the group without tuberculosis. Higher CRP quotients improved specificity at the expense of sensitivity.

**Significance:**

In high HIV prevalence settings a normal CRP could be a useful test in combination with clinical evaluation to rule out tuberculosis in ambulatory patients. Point-of-care CRP should be further evaluated in primary care clinics.

## Introduction

The HIV epidemic has substantially increased the incidence of tuberculosis, and especially smear-negative tuberculosis (SNTB), in high prevalence resource-limited settings [1]–[3]. Tuberculosis is a curable infection yet remains the leading cause of death in sub-Saharan Africa [4]–[6], in part due to delayed diagnosis. New diagnostic tests for tuberculosis are urgently required and are under evaluation, but currently sputum microscopy is the only widely available diagnostic test for tuberculosis [7].

C-reactive protein (CRP) is a non-specific acute phase serum protein that is elevated in HIV seropositive and seronegative patients with tuberculosis [8]–[15]. Recently, CRP has been proposed as a candidate biomarker for active infection with *Mycobacterium tuberculosis* [16]. Point-of-care CRP testing has been shown to be of use in the clinical evaluation of respiratory tract infections in adults [17]–[19] and in the evaluation of fever in children [20]–[22]. Additionally, an elevated point-of-care CRP has been used as an indication to initiate antibiotic therapy [18]. The diagnostic utility of CRP in smear-negative tuberculosis suspects in a high HIV prevalence setting is unknown.

We evaluated serum CRP in sputum smear-negative tuberculosis suspects in the Umgungundlovu District, KwaZulu-Natal, South Africa.

## Methods

### Ethics statement

The study was approved by the Biomedical Research Ethics Committees of the University of KwaZulu-Natal and the University of Cape Town, and by the KwaZulu-Natal Department of Health. All participants gave written informed consent prior to enrolment.

### Study setting

Tuberculosis suspects were enrolled between June 2005 and February 2007 from primary care clinics in the uMgungundlovu District, KwaZulu-Natal, South Africa. The district is representative of the high tuberculosis and HIV burden in southern Africa: in 2007 the HIV prevalence in public sector antenatal clinics was 40.4%, and the estimated 2006 annual incidence of tuberculosis was 1,094 cases/100,000 [23], [24].

### Study population

Consecutive adults older than 18 years who gave informed consent were eligible for inclusion if they were being worked up by the primary care clinic as TB suspects. Inclusion criteria were one or more symptoms compatible with tuberculosis for >2 weeks (cough, weight loss, loss of appetite, haemoptysis, fevers and chills, drenching night sweats, fatigue, chest pain, haemoptysis, shortness of breath, swollen lymph nodes, abdominal swelling), and two or more sputum smears negative for acid-fast bacilli (AFB) or unable to produce sputum. Exclusion criteria were: missing baseline CRP, not completing eight weeks of follow-up, Karnofsky performance score <40, tuberculous meningitis, *Pneumocystis jirovecii* pneumonia, more than one week of antitubercular therapy, less than three months of antiretroviral therapy, or a flouroquinolone within the past 6 months.

### Study procedures

Eligible participants were assessed for evidence of SNTB using a standardized protocol that included chest radiograph in all cases, and abdominal and pericardial ultrasound if indicated. These clinical and radiological data were used to assign participants either to antitubercular therapy or observation using pre-specified criteria. Participants were assigned to antitubercular therapy if clinicians identified a focal process compatible with tuberculosis either on physical examination or radiographic imaging using modifications of previously validated case definitions [9].

At enrolment at least two relevant specimens were taken for mycobacterial culture. Sputum induction with hypertonic saline and an ultrasonic nebuliser was performed in all participants. All specimens sent for mycobacterial culture were stained for acid-fast bacilli using fluorescent microscopy and cultured in liquid culture media (BACTEC™ MGIT™ BD, Sparks, MD). Positive cultures were identified as *Mycobacterium tuberculosis* using the niacin test. All laboratory investigations were performed in accordance with manufacturers' instructions at accredited laboratories by technicians registered with the Health Professions Council of South Africa. External quality assurance for sputum smears was routinely undertaken by the National Health Laboratory Service. Technicians evaluating specimens for acid-fast bacilli and positive mycobacterial cultures did not have access to CRP results. Serum CRP was measured with the Olympus AU640 (normal range 0–8 mg/L) for the initial 182 participants; and the Dade Dimension RXL (normal range 0–5 mg/L) for the subsequent 182 participants.

Participants were reviewed 2, 4 and 8 weeks after enrolment. Patients in the observation group who had a positive AFB smear or mycobacterial culture, or who clinically deteriorated during follow-up were started on antitubercular therapy and reviewed for an alternative diagnosis. Participants in the treatment group who deteriorated were reviewed for an alternative diagnosis. At each visit data were collected on weight, haemoglobin concentration, Karnofsky performance score and symptoms score (number of tuberculosis-related symptoms rated ‘much better’ or ‘resolved’ dividing by the total number of tuberculosis-related symptoms present at enrolment).

### Diagnosis of tuberculosis

Participants were classified into one of three groups: i) confirmed tuberculosis (at least one clinical specimen culture-positive for *Mycobacterium tuberculosis* or AFB with granulomata on histology); ii) possible tuberculosis (focal disease process compatible with tuberculosis detected on clinical examination or imaging, or a wasting syndrome, and clinical response to anti-tubercular therapy); iii) no tuberculosis (culture-negative, without a focal process or wasting and with a clinically stable course over an eight-week observation period, or an alternative diagnosis made, or no response to empiric antitubercular therapy). Research clinicians had access to the CRP result only after the decision whether or not to initiate antitubercular therapy had been made. Clinical response to antitubercular therapy was defined as meeting two or more criteria at week 8: i) weight gain >5%; ii) haemoglobin increase >1.0 g/dL; iii) increase in Karnofsky score >20 (or >10 if baseline score was 80 or 90); and iv) symptom score >0.5 (i.e., at least half of the symptoms much better or resolved). We have previously evaluated these criteria [9].

### Data analysis

Data were entered into a Microsoft Access database and analysed using Analyse-it for Microsoft Excel (version 2.11). In order to correct for the differing normal ranges CRP results were expressed as the quotient of the absolute CRP value divided by the upper limit of normal for the assay. In order to determine the most useful cut-off for the CRP quotient the performance characteristics were calculated for measurements that were either normal or raised (CRP quotient >1× upper limit of normal [ULN]), and either greater than or less than 2.5×, 5× and 10× ULN. Categorical data were compared between different groups using the Pearson chi-squared test, and odds ratio using Fisher's exact test. Accuracy of probability estimates were determined by calculating ninety five percent confidence intervals (95% CI). Distribution of continuous data was determined using the Shapiro-Wilk test, difference in medians between two groups using the Mann-Whitney test, and difference in medians between more than two groups using the Kruskal-Wallis test.

## Results

### Cohort characteristics

**Figure 1:**
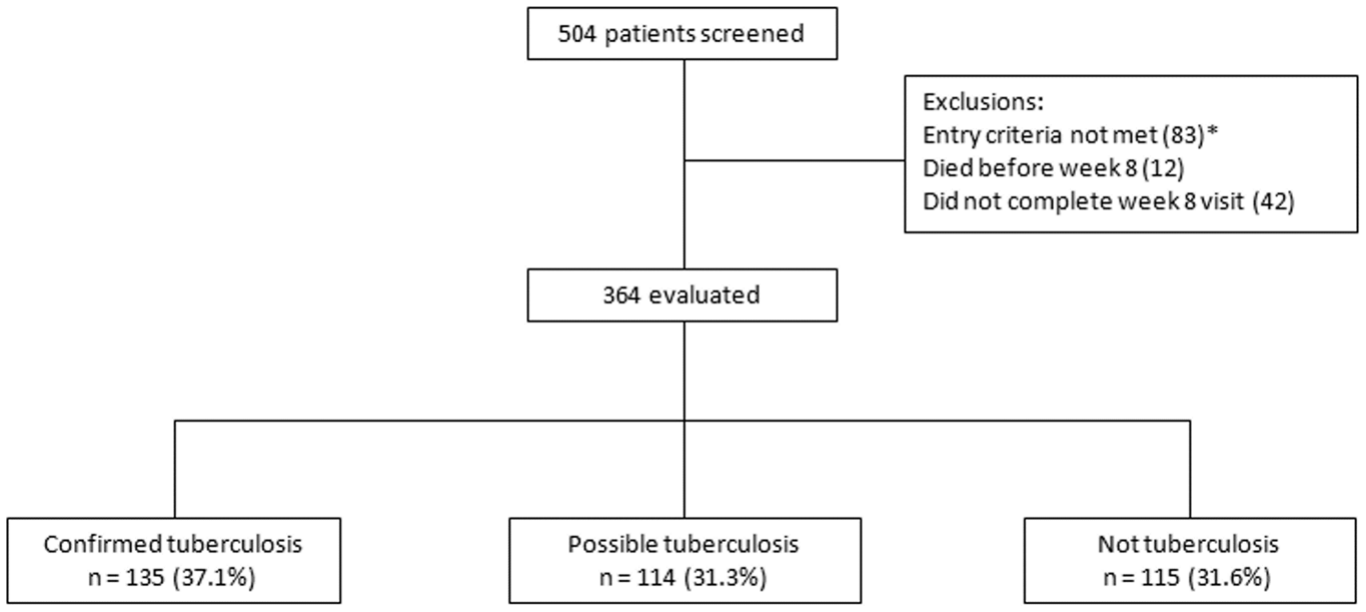
Participant flow chart and final diagnosis. * Reasons for exclusion (n) Not able to attend for regular review - determined during screening visit (28) No active symptoms (17) Alternative medical diagnosis made at screening (14) Karnofsky Score <40 (5) Pneumocystis pneumonia (4) Informed consent not obtained (3) Sputum smear positive (3) Already on antitubercular therapy (3) Other (6).

Five hundred and four patients were screened, and 364 participants evaluated. Participant flow and final diagnosis is summarized in Figure 1. The clinical characteristics of the participants are shown in Table 1. Two hundred (55%) participants were HIV seropositive, 39 (11.%) HIV seronegative, and 125 (34%) declined an HIV test. The median CD4 T-lymphocyte count was 143 cells/µL (interquartile range [IQR] 78; 248) in the 136 HIV seropositive participants who had the test during the eight week follow-up period.

**Table 1:** Participant characteristics (n = 364).

Characteristic	
Age, median (IQR), years	34.4 (29.3–42.1)
Men, n (%)	203 (57.5)
HIV seropositive, n (%) [of 239 who tested]	200 (83.7)
Prior tuberculosis treatment, n (%)	98 (26.9)
Cough >2 weeks, n (%)	341 (93.7)
Weight loss, n (%)	277 (76.1)
Anorexia, n (%)	256 (70.3)
Drenching night sweats, n (%)	248 (68.1)
Fatigue, n (%)	232 (63.7)
Fever and chills, n (%)	219 (60.2)
Chest pain, n (%)	207 (56.9)
Dyspnoea, n (%)	145 (39.8)
Haemoptysis, n (%)	40 (11.0)
Lymph node swelling, n (%)	34 (9.3)
Abdominal swelling, n (%)	4 (1.1)
Received antibiotic within 2 weeks of enrolment, n (%)	261 (71.7)
Karnofsky Performance Score, median (IQR)	70 (60–80)
Respiratory rate, median (IQR) breaths/minute	24 (20–30)
Temperature, median (IQR) °C	36.8 (36.0–37.5)
Resting heart rate, median (IQR)	100 (84–120)
Weight, median (IQR) kg	57.5 (50.6–64.8)
Body mass index, median (IQR) kg/m^2^	21.2 (18.9–23.6)
Haemoglobin, mean (SD) g/dL	11.1 (2.3)

### Tuberculosis diagnosis

One hundred and thirty five (37%) participants were classified as having confirmed tuberculosis (132 on culture and three on histology), and 114 (31%) had possible tuberculosis (including two who were originally assigned to the observation arm, deteriorated, and subsequently responded to antitubercular therapy). One hundred and fifteen participants (32%) were classified as not having tuberculosis: 68 remained stable without antitubercular therapy for eight weeks, 26 had no response to antitubercular therapy criteria, and 3 deteriorated on antitubercular therapy and another diagnosis was made. Of the 249 participants diagnosed with confirmed or possible tuberculosis 112 (45%) had pulmonary disease, 63 (25%) had both pulmonary and extrapulmonary disease and 74 (30%) had extrapulmonary disease only. Five (1.4%) of the participants were on antiretroviral therapy for >3 months (two with confirmed tuberculosis and three without tuberculosis).

### CRP quotient by tuberculosis diagnosis

The median CRP quotient in the confirmed tuberculosis group was 15.4 (IQR 7.2; 23.3), 5.8 (IQR 1.4; 16.0) in the group with possible tuberculosis, and 0.7 (IQR 0.2; 2.2) in the group without tuberculosis (*p*<0.0001). Three (2%), 23 (20%) and 68 (59%) participants had a normal CRP in these three groups respectively (*p*<0.0001).

Of the 135 participants diagnosed with confirmed tuberculosis 116 (86%) were diagnosed with a focal disease process consistent with tuberculosis: 36 had pulmonary disease; 33 had both pulmonary and extrapulmonary disease; and 47 had extrapulmonary disease. The median CRP quotient in these three groups was 12.4 (IQR 7.1–17.4), 18.2 (IQR 12.6–27.3) and 21.6 (IQR 9.3–27.6) (*p* = 0.01). The 19 participants with confirmed tuberculosis that was not diagnosed during the initial clinical evaluation had a median CRP quotient of 7.0 (IQR 2.4–16.2) compared to the median CRP quotient of 15.9 (IQR 9.4–24.5) in the 116 participants diagnosed with a focal process (*p* = 0.0003).

**Figure 2:**
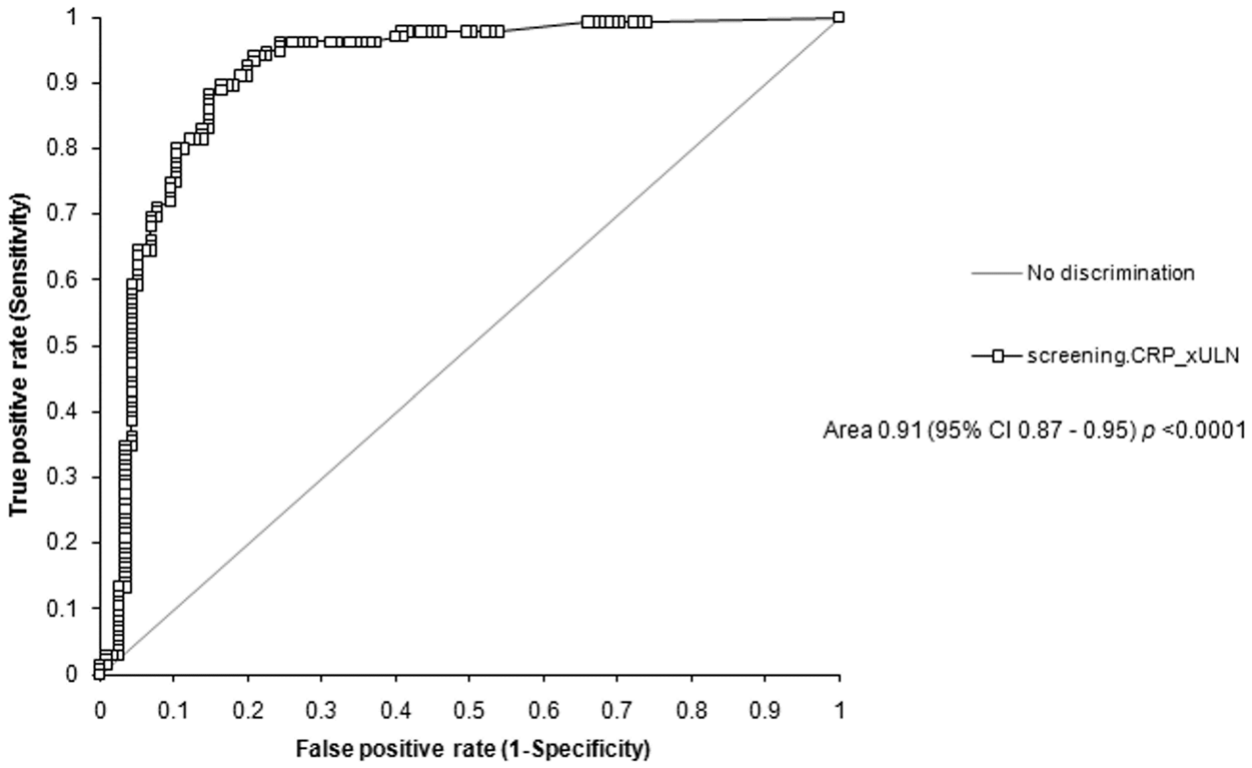
Receiver operating curves and sensitivity/specificity decision plots. A and B comparing participants with confirmed tuberculosis vs. those with no tuberculosis (n = 250); C and D combined confirmed and possible tuberculosis vs. those with no tuberculosis (n = 364); and E and F confirmed tuberculosis vs. those with possible tuberculosis and no tuberculosis.

Receiver operating curve characteristics and sensitivity/specificity decision plots for the group with confirmed tuberculosis and for the group with confirmed and possible tuberculosis are shown in Figure 2. Performance of the screening CRP quotient at various levels above the upper limit of normal are shown in Tables 2–4. The CRP quotient above the upper limit of normal had sensitivity 0.98 (95% CI 0.94; 0.99), specificity 0.59 (95% CI 0.50; 0.68), positive predictive value 0.74 (95% CI 0.67; 0.80), negative predictive value 0.96 (95% CI 0.88; 0.99), and diagnostic odds ratio 63.7 (95% CI 19.1; 212.0) in the confirmed tuberculosis group compared with the group without tuberculosis (Table 2). The positive predictive value was highest in the analyses that combined the confirmed and possible tuberculosis group (Table 3). Sensitivity and negative predictive value were maintained at the expense of the specificity and positive predictive value when the confirmed tuberculosis group was compared with the combined possible tuberculosis and no tuberculosis groups (Table 4). Higher CRP quotients improved specificity at the expense of sensitivity.

**Table 2:** Performance of CRP as a screening test: Confirmed tuberculosis vs. those with no tuberculosis (n = 250).

CRP quotient	Sensitivity	Specificity	Positive likelihood ratio	Negative likelihood ratio	Diagnostic odds ratio	Positive predictive value	Negative predictive value
	(95% CI)	(95% CI)	(95% CI)	(95% CI)	(95% CI)	(95% CI)	(95% CI)
>1×ULN	0.98	0.59	2.39	0.04	63.7	0.74	0.96
	(0.94; 0.99)	(0.50; 0.68)	(2.29; 2.49)	(0.02; 0.07)	(19.1; 212.0)	(0.67; 0.80)	(0.88; 0.99)
≥2.5×ULN	0.95	0.77	4.19	0.07	62.6	0.83	0.93
	(0.90; 0.98)	(0.69; 0.85)	(3.89; 4.52)	(0.05; 0.09)	(26.0; 150.5)	(0.76; 0.89)	(0.86; 0.97)
≥5×ULN	0.88	0.85	5.96	0.14	42.9	0.87	0.86
	(0.81; 0.93)	(0.77; 0.91)	(5.30; 6.71)	(0.12; 0.16)	(20.6; 89.2)	(0.81; 0.92)	(0.78; 0.92)
≥10×ULN	0.69	0.93	9.90	0.33	29.6	0.92	0.72
	(0.64; 0.77)	(0.87; 0.97)	(7.68; 12.77)	(0.32; 0.35)	(13.2; 66.3)	(0.85; 0.96)	(0.64; 0.79)

**Table 3 pone-0015248-t003:** Performance of CRP as a screening test: Combined group of confirmed or possible tuberculosis vs. those with no tuberculosis (n = 364).

CRP quotient	Sensitivity	Specificity	Positive likelihood ratio	Negative likelihood ratio	Diagnostic odds ratio	Positive predictive value	Negative predictive value
	(95% CI)	(95% CI)	(95% CI)	(95% CI)	(95% CI)	(95% CI)	(95% CI)
>1×ULN	0.90	0.59	2.19	0.18	12.4	0.83	0.72
	(0.85;0.93)	(0.50; 0.68)	(2.1; 2.3)	(0.16; 0.19)	(7.1; 21.5)	(0.77; 0.87)	(0.62; 0.81)
≥2.5×ULN	0.81	0.77	3.59	0.24	14.7	0.89	0.65
	(0.76; 0.86)	(0.69; 0.85)	(3.32; 3.88)	(0.23; 0.25)	(8.6; 25.2)	(0.84; 0.92)	(0.57; 0.73)
≥5×ULN	0.71	0.85	4.84	0.33	14.4	0.91	0.58
	(0.65; 0.77)	(0.77; 0.91)	(4.29; 5.45)	(0.32; 0.34)	(8.1; 25.9)	(0.86; 0.95)	(0.50; 0.65)
≥10 u ULN	0.53	0.93	7.56	0.51	14.8	0.94	0.48
	(0.46; 0.59)	(0.87; 0.97)	(5.84; 9.79)	(0.50; 0.52)	(6.9; 31.7)	(0.89; 0.97)	(0.41; 0.54)

**Table 4:** Performance of CRP as a screening test: Confirmed tuberculosis vs. those with possible tuberculosis or without tuberculosis (n = 364).

CRP quotient	Sensitivity	Specificity	Positive likelihood ratio	Negative likelihood ratio	Diagnostic odds ratio	Positive predictive value	Negative predictive value
	(95% CI)	(95% CI)	(95% CI)	(95% CI)	(95% CI)	(95% CI)	(95% CI)
>1×ULN	0.97	0.4	1.62	0.07	22.0	0.49	0.96
	(0.92; 0.99)	(0.34; 0.47)	(1.45; 1.81)	(0.03; 0.20)	(7.8; 61.6)	(0.42; 0.55)	(0.90; 0.99)
≥2.5×ULN	0.95	0.56	2.17	0.09	23.6	0.56	0.95
	(0.90; 0.98)	(0.50; 0.63)	(1.86; 2.53)	(0.04; 0.19)	(10.5; 52.7)	(0.49; 0.63)	(0.90; 0.98)
≥5 ×ULN	0.88	0.67	2.66	0.18	14.9	0.61	0.90
	(0.81; 0.93)	(0.60; 0.73)	(2.19; 3.22)	(0.11; 0.28)	(8.3: 27.0)	(0.54; 0.68)	(0.85; 0.94)
≥10×ULN	0.69	0.80	3.43	0.39	8.8	0.67	0.81
	(0.60; 0.77)	(0.74; 0.85)	(2.59; 4.55)	(0.30; 0.50)	(5.4; 14.3)	(0.58; 0.75)	(0.76; 0.86)

The two CRP assays used showed similar performance characteristics in subgroup analyses of the confirmed tuberculosis group vs. those without confirmed tuberculosis (Figure S1).

The median CRP quotient was not affected by previous treatment for tuberculosis in the 135 participants with confirmed tuberculosis (16.5 [IQR 11.4; 21.2] and 14.2 [IQR 7.1; 23.4] in the participants with and without prior tuberculosis respectively, *p* = 0.58). Prescription of an antibiotic before enrolment did not significantly alter the median CRP quotient in subgroup analysis of participants with confirmed, possible and without tuberculosis (*p*≥0.57, data not shown).

The performance characteristics of the CRP quotient were similar when the 54 participants without week 8 data were included (15 [28%] with confirmed tuberculosis) in an intention to treat analysis (Figure S2): for those with confirmed tuberculosis versus those without confirmed tuberculosis the area under the receiver operating curve was 0.81 (95% CI 0.77; 0.85) *p*<0.0001, and sensitivity/specificity decision plot intercept rate 0.75 and CRP quotient 7.4.

### Effect of HIV status on CRP quotient

HIV status did not significantly influence the median CRP in the three diagnostic categories (Table 5). Fifty five HIV seropositive patients were diagnosed with confirmed tuberculosis and had a CD4 T-lymphocyte count result. Forty had a CD4 count of <200 cells/µL and median CRP quotient of 19.9 (IQR 12.8; 25.1), and 15 had a CD4 count ≥200 cells/µL and median CRP quotient of 8.2 (IQR 6.5; 15.1) (*p* = 0.001). Performance of CRP quotient by HIV serostatus are shown in Table S1 and S2.

**Table 5:** Influence of HIV status on screening CRP.

	HIV seropositive	Median CRP quotient (IQR)		
Tuberculosis diagnosis	n (%)	HIV seropositive(n = 200)	HIV seronegative(n = 39)	*p* for CRP comparison
Confirmed tuberculosis	79 (89)	17.7	11.4	0.17
(n = 89)		(7.7; 23.7)	(3.9; 20.8)	
Possible tuberculosis	61 (82)	4.4	6.2	0.69
(n = 74)		(1.4; 11.3)	(1.1; 20.3)	
No tuberculosis	60 (79)	0.7	1.0	0.79
(n = 76)		(0.3; 2.0)	(0.2; 1.7)	
*p* for trend	0.22	<0.0001	0.0005	

## Discussion

We have shown that CRP has high sensitivity for the diagnosis of tuberculosis in this well-defined ambulatory cohort of SNTB suspects. Importantly, the CRP quotient was not affected by HIV status, and in HIV seropositive participants with confirmed tuberculosis was significantly higher in those with advanced HIV disease. Overall, higher CRP quotients were associated with increasing specificity and positive predictive value at the cost of lower sensitivity and negative predictive values. However, the high negative predictive value of any elevated CRP (0.96 for participants with confirmed tuberculosis and 0.72 for all with tuberculosis) suggests that a normal CRP would be useful for ruling out tuberculosis.

Elevated CRP levels have been demonstrated in HIV infected patients with confirmed tuberculosis [8]–[11] and in patients with a clinical syndrome compatible with either pneumonia or tuberculosis [11]–[14]. Breen et al found that an elevated CRP detected 85% of proven tuberculosis cases in London, with a 29% HIV seroprevalence in the cohort [15]. This study develops these findings by focusing on the performance of CRP in ambulatory SNTB suspects in a resource-limited high HIV prevalence setting in sub-Saharan Africa. Recent data from Europe and the United Kingdom have demonstrated the feasibility and acceptability of using a point-of-care CRP assay in primary care settings. [18]–[22]. CRP may have a role in algorithms for the evaluation of SNTB in sub-Saharan Africa where the need for novel tuberculosis diagnostics is greatest. Screening CRP may be most effectively used in early in diagnostic algorithms in primary care settings to triage smear-negative tuberculosis suspects for onward referral for chest radiograph and clinician review.

This study has several limitations. Participant enrolment was from a single site in an area with exceptionally high prevalence of HIV and incidence of tuberculosis. The utility of CRP in the diagnosis of SNTB should be evaluated in other primary care settings in sub-Saharan Africa where the prevalence of tuberculosis and/or HIV is lower. Secondly, the research clinicians had access to the screening CRP result during the follow-up visits and this may have affected evaluation of the subjective components of the response to antitubercular therapy (Karnofsky Performance Score and symptom ratio). Third, almost all participants were not taking antiretroviral therapy at the time of the study. CRP is elevated in patients experiencing the immune reconstitution inflammatory syndrome after initiating antiretroviral therapy and the findings from this study should not be extrapolated to these patients [25]. Finally, participants in this study were adult, had a Karnofsky Score of ≥40; thus our findings cannot be extrapolated to hospitalized patients or children.

In conclusion our data indicate that CRP could have a role in sub-Saharan Africa in the evaluation of tuberculosis suspects who are sputum smear-negative. CRP has the potential to contribute to future algorithms for the primary care diagnosis of SNTB in high HIV prevalence settings in combination with clinical evaluation. The utility of point-of-care CRP in screening for SNTB should be evaluated in primary care settings.

## Supporting Information

Figure S1 **Receiver operating curves and sensitivity/specificity curves for confirmed TB vs. possible TB and not TB: comparison between the two CRP assays.** A Initial 182 participants (confirmed TB *n* = 43) using the Olympus AU640 (normal range 0–8 mg/L); B Subsequent 182 participants (confirmed TB *n* = 92) using the Dade Dimension RXL (normal range 0–5 mg/L).
(TIF)

Figure S2 **Receiver operating curves and sensitivity/specificity decision plots: intention to treat analysis (150 confirmed tuberculosis; 163 clinically diagnosed tuberculosis; 105 observed).** A and B comparing participants with confirmed tuberculosis vs. those with no tuberculosis (n = 255); C and D combined confirmed and possible tuberculosis vs. those with no tuberculosis (n = 418); and E and F confirmed tuberculosis vs. those with possible tuberculosis and no tuberculosis (n = 418).
(TIF)

Table S1 **Sensitivity and specificity for the comparison confirmed TB vs. possible TB and not TB in HIV seropositive participants (n = 200).**
(DOC)

Table S2 **Sensitivity and specificity for the comparison confirmed TB vs. possible TB and not TB in HIV seronegative participants (n = 39).**
(DOC)
