# Supplementary figures and images for: Performance of Serum C-Reactive Protein as a Screening Test for Smear-Negative Tuberculosis in an Ambulatory High HIV Prevalence Population
Source: PLoS One. 2011 Jan 10;6(1):e15248. doi: 10.1371/journal.pone.0015248 (PMC3018418; doi:10.1371/journal.pone.0015248)

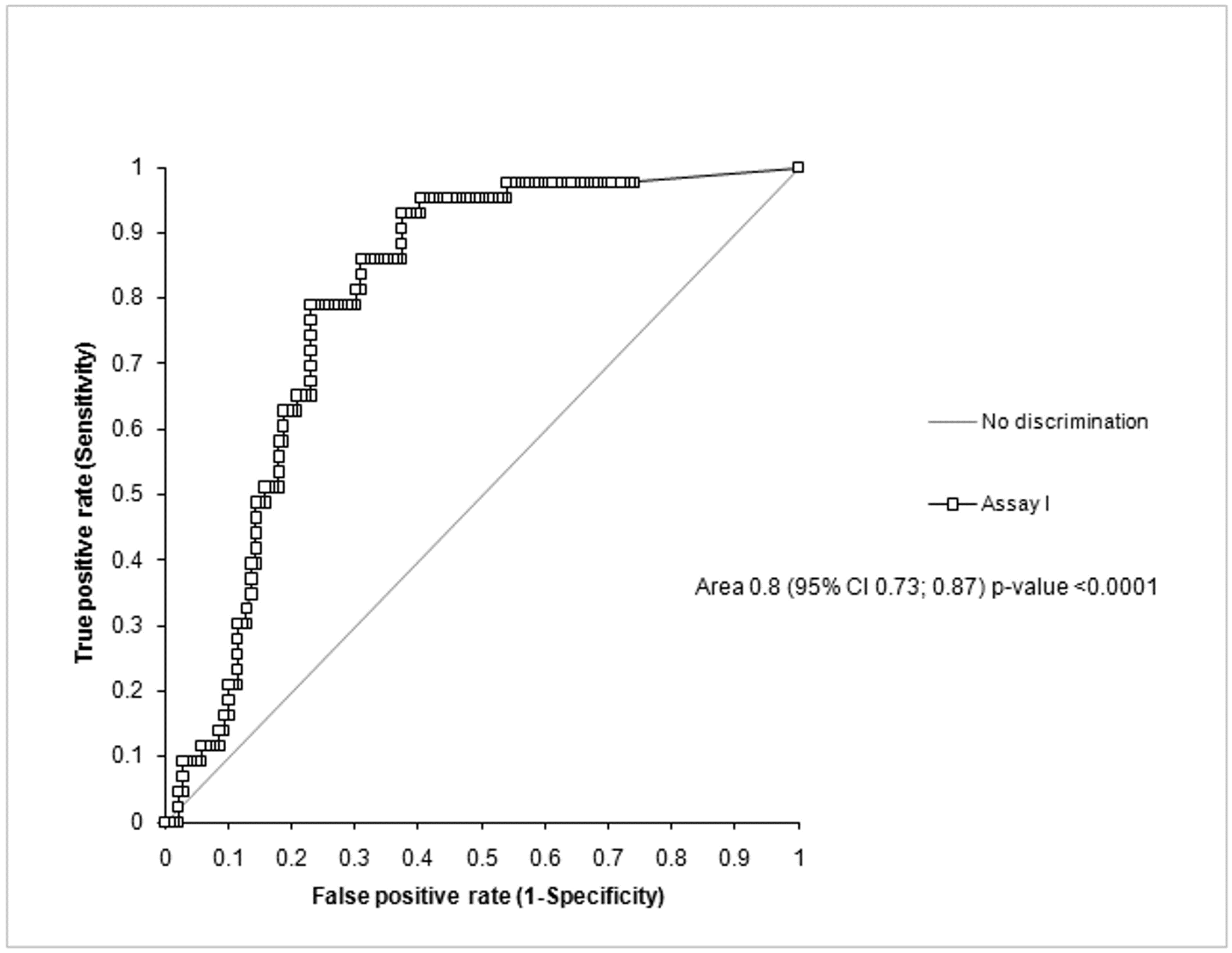

Supplement: Figure S1 — Receiver operating curves and sensitivity/specificity curves for confirmed TB vs. possible TB and not TB: comparison between the two CRP assays. A Initial 182 participants (confirmed TB n = 43) using the Olympus AU640 (normal range 0–8 mg/L); B Subsequent 182 participants (confirmed TB n = 92) using the Dade Dimension RXL (normal range 0–5 mg/L). (TIF) [file pone.0015248.s001.tif]

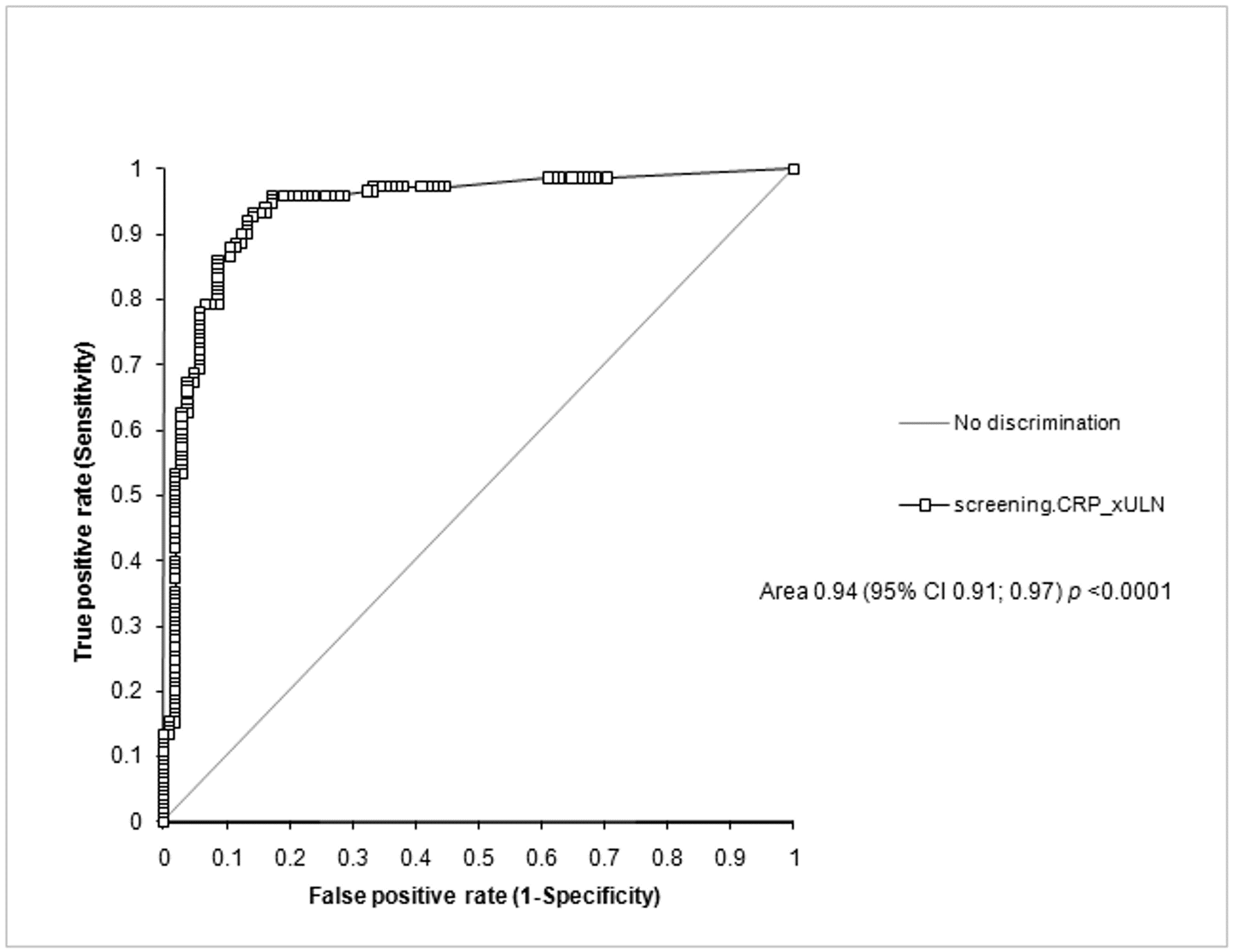

Supplement: Figure S2 — Receiver operating curves and sensitivity/specificity decision plots: intention to treat analysis (150 confirmed tuberculosis; 163 clinically diagnosed tuberculosis; 105 observed). A and B comparing participants with confirmed tuberculosis vs. those with no tuberculosis (n = 255); C and D combined confirmed and possible tuberculosis vs. those with no tuberculosis (n = 418); and E and F confirmed tuberculosis vs. those with possible tuberculosis and no tuberculosis (n = 418). (TIF) [file pone.0015248.s002.tif]
