# Supplementary material for: Performance of Serum C-Reactive Protein as a Screening Test for Smear-Negative Tuberculosis in an Ambulatory High HIV Prevalence Population
Source: PLoS One. 2011 Jan 10;6(1):e15248. doi: 10.1371/journal.pone.0015248 (PMC3018418; doi:10.1371/journal.pone.0015248)
Supplement: Table S1 — Sensitivity and specificity for the comparison confirmed TB vs. possible TB and not TB in HIV seropositive participants (n = 200). (DOC) [file pone.0015248.s003.doc]

Table S1

| CRP quotient | Sensitivity | Specificity | Positive likelihood ratio | Negative likelihood ratio | Diagnostic odds ratio | Positive predictive value | Negative predictive value |
| --- | --- | --- | --- | --- | --- | --- | --- |
|  | (95% CI) | (95% CI) | (95% CI) | (95% CI) | (95% CI) | (95% CI) | (95% CI) |
| >1 x ULN | 0.99 | 0.43 | 1.73 | 0.03 | 58.8 | 0.53 | 0.98 |
|  | (0.93; 1.00) | (0.34; 0.52) | (1.48; 2.03) | (0.004; 0.21) | (7.9; 436.5) | (0.45; 0.61) | (0.90; 1.00) |
| ≥ 2.5 x ULN | 0.95 | 0.56 | 2.17 | 0.09 | 24.0 | 0.59 | 0.94 |
|  | (0.87; 0.99) | (0.47; 0.65) | (1.76; 2.67) | (0.03; 0.24) | (8.3; 70.0) | (0.50; 0.67) | (0.86; 0.98) |
| ≥5 x ULN | 0.92 | 0.67 | 2.80 | 0.11 | 24.6 | 0.65 | 0.93 |
|  | (0.84; 0.97) | (0.58; 0.75) | (2.15; 3.63) | (0.05; 0.25) | (9.9; 61.5) | (0.55; 0.73) | (0.86; 0.97) |
| ≥10 x ULN | 0.72 | 0.81 | 3.80 | 0.34 | 11.0 | 0.71 | 0.82 |
|  | (0.61; 0.82) | (0.73; 0.88) | (2.56; 5.62) | (0.24; 0.49) | (5.6; 21.6) | (0.60; 0.80) | (0.74; 0.88) |
