# Supplementary material for: Performance of Serum C-Reactive Protein as a Screening Test for Smear-Negative Tuberculosis in an Ambulatory High HIV Prevalence Population
Source: PLoS One. 2011 Jan 10;6(1):e15248. doi: 10.1371/journal.pone.0015248 (PMC3018418; doi:10.1371/journal.pone.0015248)
Supplement: Table S2 — Sensitivity and specificity for the comparison confirmed TB vs. possible TB and not TB in HIV seronegative participants (n = 39). (DOC) [file pone.0015248.s004.doc]

Table S2

| CRP quotient | Sensitivity | Specificity | Positive likelihood ratio | Negative likelihood ratio | Diagnostic odds ratio | Positive predictive value | Negative predictive value |
| --- | --- | --- | --- | --- | --- | --- | --- |
|  | (95% CI) | (95% CI) | (95% CI) | (95% CI) | (95% CI) | (95% CI) | (95% CI) |
|  |  |  |  |  |  |  |  |
| >1 x ULN | 1.00 | 0.38 | 1.55^*^ | 0.12^*^ | 13.1 | 0.36 | 1.00 |
|  | (0.69; 1.00) | (0.21; 0.58) | (1.14; 2.11) | (0.008; 1.85) | (0.70; 245) | (0.19; 0.56) | (0.71; 1.00) |
| ≥ 2.5 x ULN | 1.00 | 0.62 | 2.49^*^ | 0.07^*^ | 33.8 | 0.48 | 1.00 |
|  | (0.69; 1.00) | (0.42; 0.79) | (1.55; 3.99) | (0.005; 1.12) | (1.8; 633) | (0.25; 0.70) | (0.81; 1.00) |
| ≥5 x ULN | 0.70 | 0.72 | 2.54 | 0.41 | 6.1 | 0.47 | 0.87 |
|  | (0.35; 0.93) | (0.53; 0.87) | (1.24; 5.19) | (0.15; 1.1) | (1.3; 29.7) | (0.21; 0.73) | (0.68; 0.97) |
| ≥10 x ULN | 0.70 | 0.82 | 4.06 | 0.36 | 11.2 | 0.58 | 0.89 |
|  | (0.35; 0.93) | (0.64; 0.94) | (1.66; 9.93) | (0.14; 0.95) | (2.1; 58.9) | (0.28; 0.85) | (0.71; 0.98) |

^*^Likelihood ratios estimated using substitution formula, with 0.5 added to all cell frequencies before calculation
